# Quantum self-consistent equation-of-motion method for computing molecular excitation energies, ionization potentials, and electron affinities on a quantum computer

**DOI:** 10.1039/d2sc05371c

**Published:** 2023-01-27

**Authors:** Ayush Asthana, Ashutosh Kumar, Vibin Abraham, Harper Grimsley, Yu Zhang, Lukasz Cincio, Sergei Tretiak, Pavel A. Dub, Sophia E. Economou, Edwin Barnes, Nicholas J. Mayhall

**Affiliations:** a Department of Chemistry, Virginia Tech Blacksburg 24061 VA USA aasthana@vt.edu nmayhall@vt.edu; b Theoretical Division, Los Alamos National Laboratory Los Alamos 87545 NM USA akumar1@lanl.gov; c Department of Chemistry, University of Michigan Ann Arbor 48109 MI USA; d Center for Integrated Nanotechnologies, Los Alamos National Laboratory Los Alamos 87545 NM USA; e Chemistry Division, Los Alamos National Laboratory Los Alamos 87545 NM USA; f Department of Physics, Virginia Tech Blacksburg 24061 VA USA; g Virginia Tech Center for Quantum Information Science and Engineering Blacksburg 24061 VA USA

## Abstract

Near-term quantum computers are expected to facilitate material and chemical research through accurate molecular simulations. Several developments have already shown that accurate ground-state energies for small molecules can be evaluated on present-day quantum devices. Although electronically excited states play a vital role in chemical processes and applications, the search for a reliable and practical approach for routine excited-state calculations on near-term quantum devices is ongoing. Inspired by excited-state methods developed for the unitary coupled-cluster theory in quantum chemistry, we present an equation-of-motion-based method to compute excitation energies following the variational quantum eigensolver algorithm for ground-state calculations on a quantum computer. We perform numerical simulations on H_2_, H_4_, H_2_O, and LiH molecules to test our quantum self-consistent equation-of-motion (q-sc-EOM) method and compare it to other current state-of-the-art methods. q-sc-EOM makes use of self-consistent operators to satisfy the vacuum annihilation condition, a critical property for accurate calculations. It provides real and size-intensive energy differences corresponding to vertical excitation energies, ionization potentials and electron affinities. We also find that q-sc-EOM is more suitable for implementation on NISQ devices as it is expected to be more resilient to noise compared with the currently available methods.

## Abstract 2

We present a new hybrid quantum algorithm to estimate molecular excited and charged states on near-term quantum computers following any VQE-based ground state estimation.

## Graphical abstract

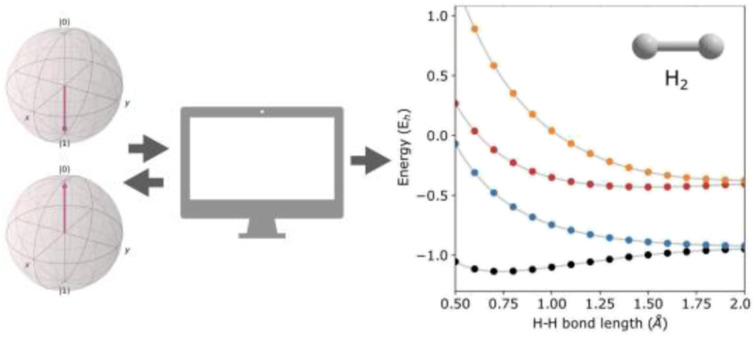

## 1. Introduction

Quantum chemistry is expected to be one of the first areas which can have demonstrable quantum advantages in the near term.^1–8^ This is due to the fact that the computational effort required for exact evaluation of electron correlation on a classical computer-whose accurate calculation is essential for a reliable comparison with experimental values-scales factorially with the number of molecular orbitals. This unfavourable scaling is expected to reduce drastically when wavefunctions are instead prepared on quantum devices.

For estimation of molecular ground-state properties on noisy intermediate-scale quantum (NISQ) era devices, variational quantum eigensolver (VQE) based algorithms have gained popularity due to their relatively low circuit depth and resilience to noise.^9,10^ This has led to a series of successful demonstrations involving the computation of molecular ground-state energies of small molecules on present-day quantum devices and simulators.^4,6,11–22^ However, estimation of just the molecular ground-state energy is not sufficient for describing many interesting chemical processes that involve electronic excitations in some form.^23^ For example, accurate modelling of chemical phenomena such as photochemical reactions, catalytic processes involving transition metal complexes, photosynthesis, solar cell operation, *etc.* requires an accurate simulation of both molecular ground and excited states. The electronically excited states of such systems are generally strongly correlated and hence, require the use of sophisticated quantum chemical theories for their accurate description. A number of methods have been developed in this regard in the last few decades.^24–32^ The equation-of-motion coupled-cluster (EOM-CC)^26^ approach, originally developed by Stanton and Bartlett, is a popular example that is routinely used to calculate molecular excited-state properties such as excitation energies and transition dipole moments.^33–37^ EOM-CC has also been extended to calculate energies required to add or remove electrons from the ground-state electronic configuration.^38–42^ For example, IP-EOM-CC^38,40^ and EA-EOM-CC^39^ approaches have been developed which can compute accurate vertical ionization potentials (IPs) and vertical electron affinities (EAs), respectively. IPs/EAs are defined as the difference in energy between the ground state and the states obtained by a single electron detachment/attachment process. Some of the other advantages associated with the EOM-CC formalism are its theoretical rigour, the accuracy and correct scaling behavior of energy differences computed, and the ability to systematically improve the results. However, standard quantum chemistry methods such as EOM-CC sometimes face challenges in a quantitative determination of excited states and their properties, notably for same-symmetry conical intersections^43–46^ and when the ground state has a prominent multi-reference character.^47–50^ Since VQE algorithms are expected to provide accurate ground-state wavefunctions, even in the case of strongly correlated systems, NISQ era devices can help address these challenging problems with practical computational expenses.

We would like to note that methods for the estimation of molecular excited states on a quantum computer based on other popular quantum algorithms have also been proposed. A number of approaches are based on the quantum phase estimation algorithm^51–57^ with new developments for efficient implementation on quantum computers.^58–60^ Methods based on Krylov subspace diagonalization^61,62^ and quantum annealing^63,64^ have also been proposed. While these methods are theoretically exact (in the absence of any noise) and expected to provide a significant computational advantage over exact treatment on a classical computer, they will mostly be useful in fault-tolerant quantum computing and not suitable for NISQ era quantum computers due to their high quantum resource requirements and low tolerance to noise.

Significant effort has been made in developing methods for the calculation of molecular excitation energies within the framework of VQE in the last few years. These techniques can be broadly classified into circuit optimization and diagonalization-based approaches. In the former approach, optimal parametrized circuits are obtained for every excited state, usually by minimizing a cost function involving energies of one or multiple excited states. Subspace-search VQE (SS-VQE),^65^ orthogonal state reduction variational eigensolver (OSRVE),^66^ variational quantum deflation (VQD)^67,68^ and the folded spectrum method^4^ are some examples. These approaches, however, generally require increased quantum resources, specifically the gate depth. This makes them challenging for near term applications. Moreover, there is no guarantee for them to find the entire spectrum when the states are close in energy to one another. On the other hand, diagonalization-based approaches use a classical computer to diagonalize the Hamiltonian in a subspace and can provide several excited states simultaneously. In this regard, methods such as quantum Krylov subspace expansion,^61,69,70^ quantum subspace expansion (QSE),^71–74^ and the quantum equation-of-motion (qEOM)^75^ have been developed recently. QSE has had significant success in the last few years and has also been extended to capture the missing correlation in large virtual orbital spaces.^73,76^ However, it requires an estimate of higher than 2-body reduced density matrices (RDMs), prompting the use of cumulant approximations^73^ inspired by developments in quantum chemistry.^31,32,77^ Furthermore, a significant drawback of the QSE approach is the lack of size-intensivity of the computed excitation energies. The property of size-intensivity ensures correct scaling of excitation energies computed by a method with increasing size of the system. The violation of this property can lead to errors and even non-physical predictions; for instance, the QSE excitation energies of a “super molecule” consisting of two non-interacting systems are not guaranteed to be the same as the excitation energies of the two systems calculated separately (see Fig. 7). This may become a severe limitation when QSE is applied to larger systems in the future and the underlying ground-state wavefunction is imprecise.

In search of a size-intensive alternative, the EOM formalism based qEOM method was proposed by Ollitrault *et al.*^75^ for electronic excitation energies (EEs). The qEOM method provides good agreement for EEs with the exact results obtained by the full configuration interaction (FCI) method. However, the qEOM formalism (in ref. 75) does not necessarily satisfy the vacuum annihilation condition (VAC), also known as the killer condition,^78,79^ which ensures that the ground-state wavefunction cannot be de-excited. This may result in the appearance of large errors when the formalism is extended to calculate properties such as IPs and EAs. Moreover, the qEOM method, just like QSE, requires higher-body RDMs which significantly increases the measurement challenges.

In this work, we propose a generally applicable EOM-based formalism for the calculation of molecular properties such as EEs, IPs, and EAs following a VQE ground-state calculation on a quantum computer. Our formalism, which we refer to as q-sc-EOM, satisfies the VAC; produces size-intensive and real energy differences between the ground state and the excited states/charged states; does not involve measurements of higher than 2-body RDM-type quantities; is expected to be more resilient to noise than the current diagonalization-based state-of-the-art methods.

This paper is organized as follows: Section 2.1. discusses the theoretical formalism of q-sc-EOM using self-consistent operators, while the implementation details and circuit design are explained in Section 2.2. Section 3. provides the computational details for the simulation data in this paper. In Section 4., we discuss the results obtained in this work. Specifically, Section 4.1. analyses the performance of the q-sc-EOM method in calculating EEs, IPs, and EAs of H_2_, LiH, and H_2_O molecules, while Section 4.2. compares the performance of the q-sc-EOM method with those of the QSE and qEOM formalisms. Finally, the key conclusions from the paper are summarized in Section 5.

## 2. Theory

The excitation energy of a given excited state can be obtained by the action of the commutator of the Hamiltonian and the corresponding excitation operator acting on the exact ground-state wavefunction. For an arbitrary *k*th excited state, this can be expressed as1
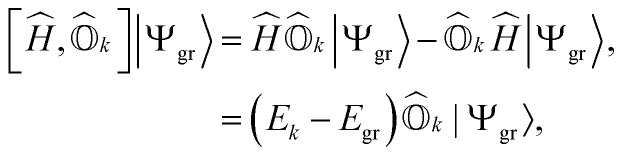
where |*Ψ*_gr_〉 is the ground-state wavefunction and *E*_gr_ and *E*_*k*_ refer to the energies of the ground and the *k*th excited state, respectively. *Ĥ* is the molecular Hamiltonian, which in the second quantization formalism can be written as2
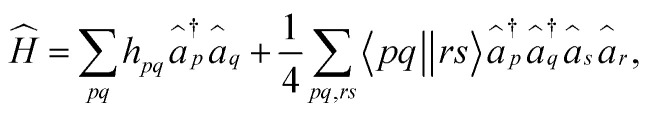
where *h*_*pq*_ and 〈*pq*‖*rs*〉 are the one- and two-electron elements of the Hamiltonian, respectively. *â*^†^_*p*_ and *â*_*p*_ refer to the fermionic creation and annihilation operators (with respect to physical vacuum), respectively. Following common notations, here, we use indices {*p*, *q*, *r*, *s*…} for arbitrary molecular orbitals while {*a*, *b*, …} and {*i*, *j*, …} refer to unoccupied and occupied orbitals, respectively in the Hartree–Fock (HF) wavefunction. The state-transfer operator 
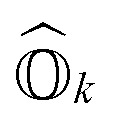
 is defined as3
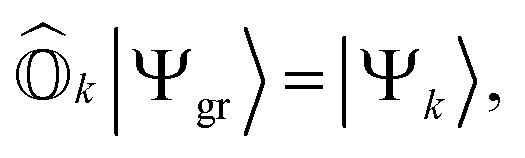
where |*Ψ*_*k*_〉 is the wavefunction of the *k*th excited state. These operators should ensure an important property, referred to as vacuum annihilation or killer condition, which expresses that the ground state cannot be de-excited:^78–83^4
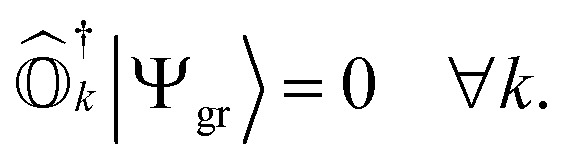
In the case of exact operators 
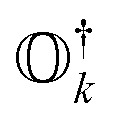
 and an exact ground-state (Ψ_gr_), the above equation can be reformulated as5
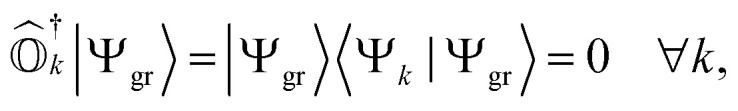
where 
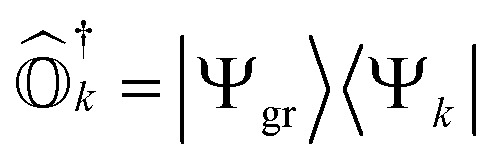
. It can be seen that this condition is automatically satisfied for exact state-transfer operators acting on an exact ground-state due to the orthogonality of eigenstates. However, one needs to ensure that the VAC is satisfied when approximate state-transfer operators are used.^78,83^ The state-transfer operators that satisfy the VAC are referred to as “self-consistent” operators.^78^

This work utilizes the framework of unitary coupled-cluster (UCC) theory, where the ground-state wavefunction is given by

6 |*Ψ*_UCC_〉 = *e*^*

<svg xmlns="http://www.w3.org/2000/svg" version="1.0" width="16.000000pt" height="16.000000pt" viewBox="0 0 16.000000 16.000000" preserveAspectRatio="xMidYMid meet"><metadata>
Created by potrace 1.16, written by Peter Selinger 2001-2019
</metadata><g transform="translate(1.000000,15.000000) scale(0.015909,-0.015909)" fill="currentColor" stroke="none"><path d="M480 840 l0 -40 -40 0 -40 0 0 -40 0 -40 40 0 40 0 0 40 0 40 40 0 40 0 0 -40 0 -40 40 0 40 0 0 40 0 40 -40 0 -40 0 0 40 0 40 -40 0 -40 0 0 -40z M240 520 l0 -40 -40 0 -40 0 0 -80 0 -80 -40 0 -40 0 0 -120 0 -120 40 0 40 0 0 -40 0 -40 160 0 160 0 0 40 0 40 40 0 40 0 0 40 0 40 40 0 40 0 0 120 0 120 -40 0 -40 0 0 40 0 40 80 0 80 0 0 40 0 40 -240 0 -240 0 0 -40z m240 -80 l0 -40 40 0 40 0 0 -80 0 -80 -40 0 -40 0 0 -40 0 -40 -40 0 -40 0 0 -40 0 -40 -80 0 -80 0 0 40 0 40 -40 0 -40 0 0 40 0 40 40 0 40 0 0 80 0 80 40 0 40 0 0 40 0 40 80 0 80 0 0 -40z"/></g></svg>

*^|*Ψ*_0_〉,

where |*Ψ*_0_〉 is the HF wavefunction and *e*^**^ is a unitary operator. An approximate form of *σ̂*, which is a cluster operator, can be written using single and double excitations as7
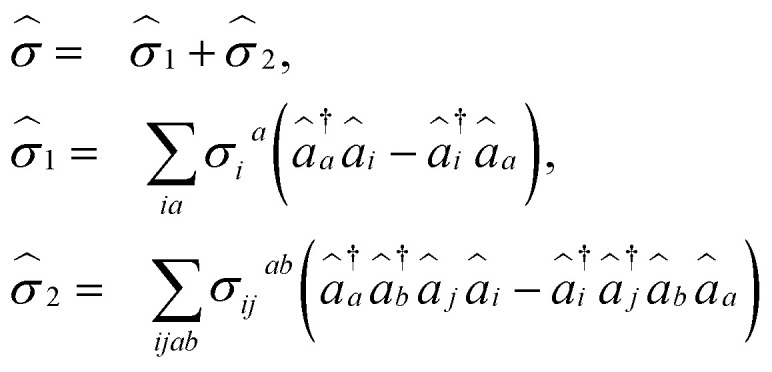
where *σ*_*i*_^*a*^ and *σ*_*ij*_^*ab*^ are amplitudes for the associated excitations. In order to capture the dominant electron-correlation effects contributing to electronic excitations, an excitation manifold can be constructed by including all possible single and double excitations and de-excitations, represented by {*Ĝ*^†^_*I*_}∪{*Ĝ*_*I*_}. Here, *Ĝ*_*I*_ can refer to any single (*â*^†^_*a*_*â*_*i*_) or double (*â*^†^_*a*_*â*^†^_*b*_*â*_*j*_*â*_*i*_) excitation operator. However, to satisfy the VAC, the excitation operator manifold is rotated, forming the self-consistent manifold {*Ŝ*^†^_*I*_}∪{*Ŝ*_*I*_}, where

8 *Ŝ*_*I*_ = *e*^*σ̂*^*Ĝ*_*I*_*e*^*−σ̂*^.

Similar excitation manifolds can also be constructed using particle number non-conserving excitation operators that are needed for computation of IPs and EAs. This technique was developed by Mukherjee and co-workers in ref. 78 and 84. This self-consistent operator manifold can now be used to develop excited-state methods that satisfy the VAC.

Following eqn (8), the state-transfer operator for electronic excitations, 
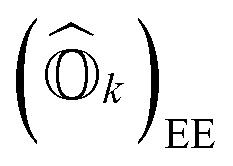
, can now be written as a linear combination of all possible operators from the self-consistent excitation manifold, given by9
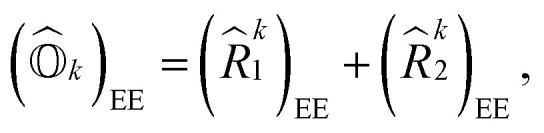
where (*R̂*^*k*^_1_)_EE_ and (*R̂*^*k*^_2_)_EE_ are single and double excitation operators defined as10
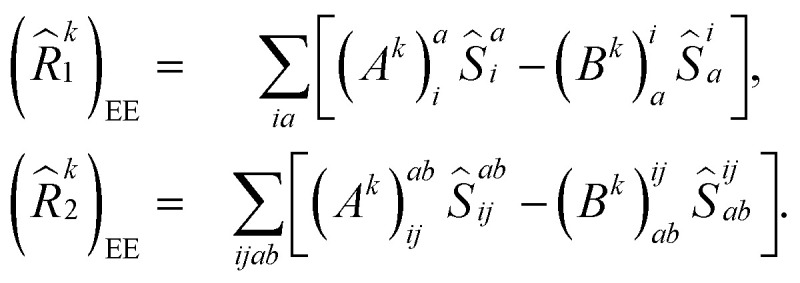
Here, (*A*^*k*^)_*I*_ and (*B*^*k*^)_*I*_^†^ are the amplitudes corresponding to the excitation (*I*) and de-excitation operators (I^†^), respectively, for the *k*th excited state. Here *I* refers to all possible single and double excitations. State-transfer operators for singly charged states can also be defined in a similar manner,11
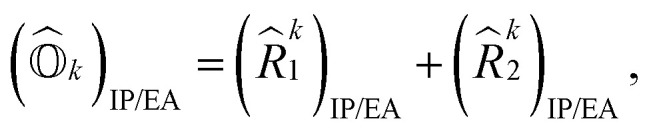
where (*R̂*^k^_1_)_IP/EA_ and (*R̂*^k^_2_)_IP/EA_ refer to the particle number non-conserving single and double excitation operators, defined as12
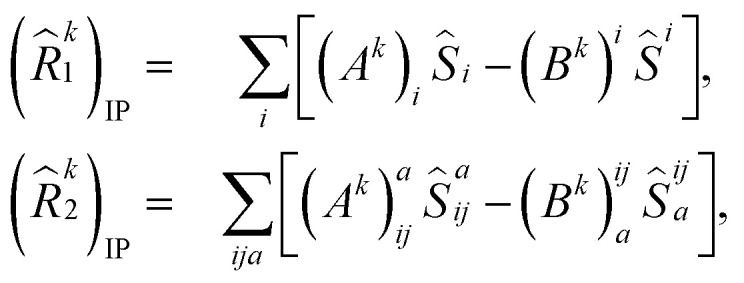


and13
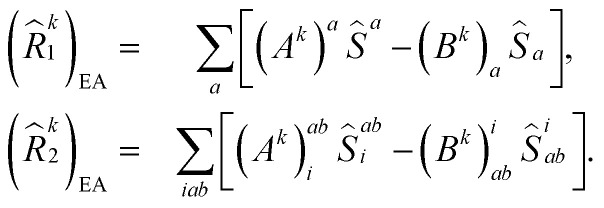


### 2.1 q-sc-EOM method

In the VQE algorithm, the unitary evolution operator *U*(*θ*) (*e*^*σ̂*^ in UCC theory) is implemented on a quantum computer using a parameterized circuit. The parameters (*θ*) of the circuit are optimized to variationally minimize the molecular energy and obtain the molecular ground state |*Ψ*_VQE_〉, such that

14 |*Ψ*_VQE_〉 = *U*(*θ*)|*Ψ*_0_〉.

By projecting eqn (1) onto the *k*th excited state wavefunction and using the state-transfer operator defined in eqn (9), the q-sc-EOM excitation energy from the ground state to the *k*th excited state (*E*_0*k*_) is given by15
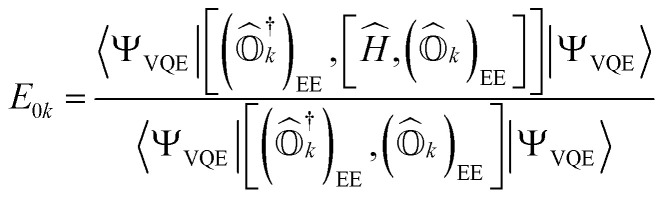


Expressions for IPs and EAs can also be derived using the associated state-transfer operators defined in eqn (11). As discussed in ref. 75, eqn (15) provides size-intensive energy differences. By inserting the expression for 
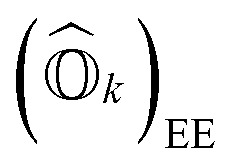
 from eqn (9) into eqn (15), it can be seen that the final equation for the excitation energy for the *k*th excited state has a parametric dependence on the amplitudes (*A*^*k*^)_*I*_ and (*B*^*k*^)^†^_*I*_, where *I* refers to all possible single and double excitations. A variational minimization of the resulting equation (δ*E*_0*k*_ = 0) with respect to these amplitudes leads to the following secular equation:16
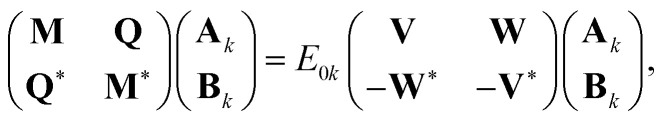
where the matrix elements of matrices **M**, **Q**, **V**, and **W** are defined as17
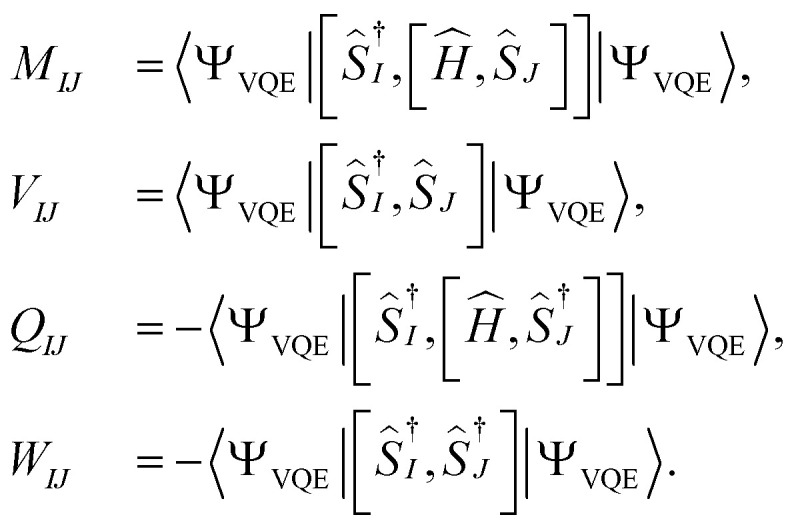


Upon careful inspection, one can see that the matrices **W** and **Q** are zero due to the use of self-consistent operators. This is a simplification (compared to the qEOM formalism of ref. 75) as it reduces the secular equation to

18 **MA**_*k*_ = *E*_0*k*_**VA**_*k*_.

The matrix elements of **M** and **V** can be further simplified as19
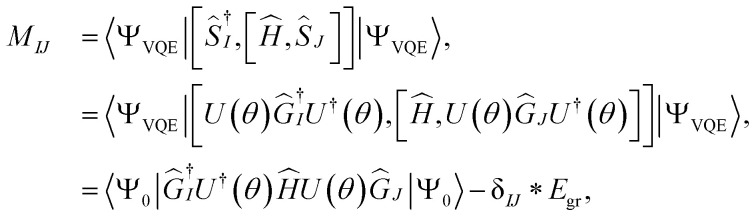


and20
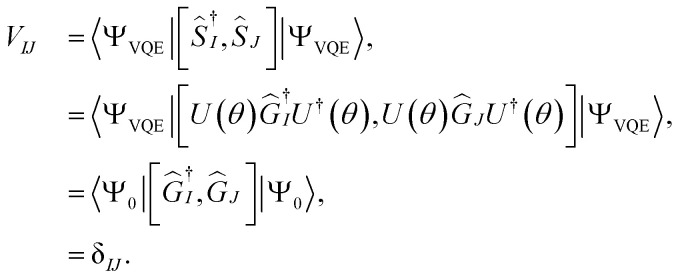


Thus, in q-sc-EOM the overlap matrix (**V**) is guaranteed to be the identity matrix and does not need to be computed on a quantum computer. It has two key benefits, namely, it leads to a Hermitian formulation for excitation energy and it converts the generalized eigenvalue equation into an eigenvalue equation.

The formalism developed so far can be written in the form of a concise eigenvalue equation as21
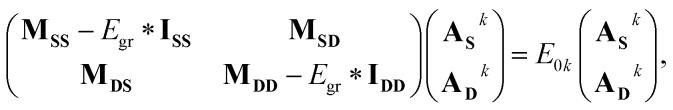
where *S* and *D* refer to single and double excitations, respectively. Thus, **M**_**SS**_ refers to the block of matrix **M** with two single excitation operators in the double commutator (see eqn (17)), while **I**_**XX**_ is an identity matrix of dimension *X*. It should be emphasized that, since the above formulation is Hermitian, the eigenvalues obtained using eqn (21) are guaranteed to be real (unlike in EOM-CCSD or qEOM).^43,75,85^ This also ensures that this formulation is free from problems related to different left and right eigenfunctions encountered in classical EOM-CC methods.^26^

Finally, each element of matrix **M** can be computed on a quantum computer using eqn (19). The resulting eigenvalue equation can then be solved classically to obtain q-sc-EOM EE values. Here, Krylov subspace based formalisms, such as the Davidson algorithm,^86–88^ can be used to obtain the excitation energies of a few low-lying excited states while avoiding the explicit evaluation and diagonalization of the **M** matrix. It should be noted that this method is closely related to UCC-based excited-state methods in quantum chemistry, and thus eqn (21) resembles the equation for EEs for UCC-based methods as derived, for example, in ref. 82 and 85.

### 2.2 Circuit design and implementation details

**Fig. 1:**
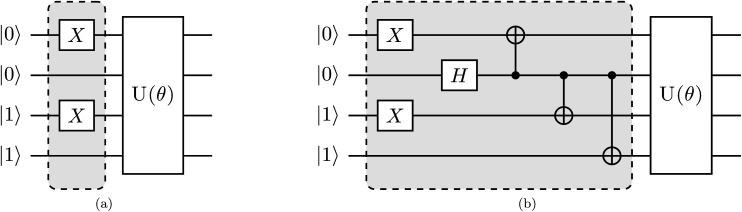
Proposed quantum circuit for the estimation of a representative element of the **M** matrix for the H_2_ molecule using (a) an excited Slater determinant as the reference state needed to compute diagonal elements and (b) an entangled state involving two excited Slater determinants as the reference required for the evaluation of off-diagonal elements.

Here, we discuss our proposed implementation of the q-sc-EOM formalism on a quantum computer. The discussion can be divided into two parts: circuit details to evaluate the diagonal and the off-diagonal elements of the matrix **M** in eqn (19). The state preparation for the diagonal elements involves the same circuit as the one optimized for the ground state, but it is now applied to a classical state that represents an excited Slater determinant. We refer to this classical state as the reference state. Thus, the circuit can be prepared in two steps: the first step is the creation of a reference state, whereas the second step involves the action of the previously optimized ground-state VQE circuit on the newly formed reference state. The molecular Hamiltonian is then measured using this prepared state, as performed for the ground-state energy evaluation. To give an example of a state preparation circuit, we consider the H_2_ molecule in the STO-3G basis and use a singly excited determinant in eqn (19). We choose the single-excitation operator for the excitation from 1s^*β*^ to 2s^*β*^ (represented by |0011〉 → |1001〉 notation in the qubit representation using the Jordan–Wigner mapping). The classical state that corresponds to such an excited Slater determinant can be created by the action of two NOT gates, as shown in the circuit in Fig. 1a. *U*(*θ*) in Fig. 1a refers to the optimized circuit prepared for the VQE ground-state evaluation, and the shaded region represents the circuit needed to create the reference state. Note that the orbitals 1s^*α*^, 1s^*β*^, 2s^*α*^, and 2s^*β*^ are mapped onto the qubits in a bottom-to-top order, such that the lowest energy orbitals are at the bottom.

The off-diagonal elements of the matrix **M** can be evaluated using an entangled state of two excited Slater determinants, as shown below. A representative off-diagonal element *M*_*I*,*J*_ can be written in terms of diagonal elements as22
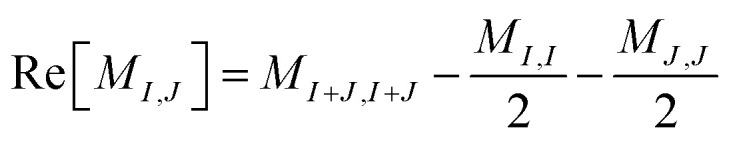
where the term *M*_*I*+*J*,*I*+*J*_ is given by23
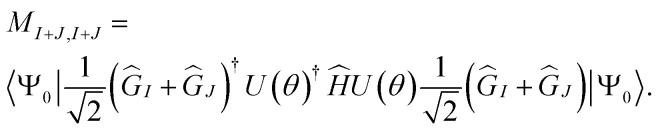


A similar expression holds for the imaginary part of *M*_*I*,*J*_. The matrix elements *M*_*I*,*I*_ and *M*_*J*,*J*_ in eqn (22) are diagonal elements that are evaluated using the method described previously (without the ground-state energy term). The element *M*_*I*+*J*,*I*+*J*_ can be evaluated using eqn (23), which involves the creation of the *I* + *J* state, application of the unitary *U*(*θ*), and finally, the measurement of the Hamiltonian using this prepared state. The state *I* + *J* is a superposition state of two classical states *I* (*Ĝ*_*I*_|Ψ_0_〉) and *J* (*Ĝ*_*J*_|Ψ_0_〉). Notice that both of these states are excited Slater determinants, which can be represented through qubit states in the computational basis. An entangled state, such as *I* + *J*, is commonly created by using an ancilla qubit (for example, see ref. 13). In the case of q-sc-EOM, we can use a simpler method to create this entanglement without adding any ancilla qubits. Being classical states, *I* and *J* are trivial to create on a quantum computer using NOT gates. An entangled state can then be created using a Hadamard gate and a series of CNOT gates. For example, the *I* state can be created using NOT gates and then transformed into the *I* + *J* state using Hadamard and CNOT gates. We can take the example of two single excitations to demonstrate this, specifically 1s^*α*^ → 2s^*α*^ and 1s^*β*^ → 2s^*β*^ (denoted as |0011〉 → |1001〉 and |0011〉 → |0110〉 in the qubit representation, respectively). Here, we need to create the entangled state 
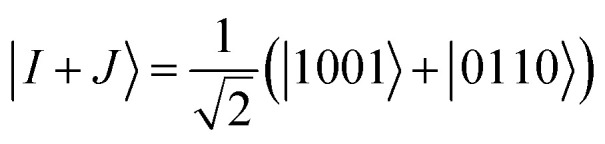
. The circuit for creating this state and then evaluating *M*_*I*+*J*,*I*+*J*_ is shown in Fig. 1b. The shaded region represents the circuit used to create the entangled |*I* + *J*〉 state. The gate sequences can be stored for each excitation operator and can be applied to the HF state at the start of the EOM procedure. A maximum of 7 CNOT gates will be needed to create any entangled state for off-diagonal terms, when using up to double excitations in eqn (19). This is a very small number compared with the total number of CNOT gates required to prepare the ground state using VQE-based algorithms. It should be noted that this proposed circuit design loses on the phase factor of excitation operators in *I* and *J* classical states, but all our numerical simulations have consistently shown that these phase factors have no effect on the computed eigenvalues of the eigenvalue equation. To preserve the phase factor, an ancilla qubit based creation of entangled *I* + *J* states could be used instead.

Regarding the resource requirements for running the q-sc-EOM method on a quantum computer, the number of qubits required for the evaluation of each matrix element is the same as that required for the computation of the ground state. The circuit that is implemented in the ground-state VQE calculation remains unchanged in the generation of the EOM matrix elements as well. The only difference is that the reference state is changed from the HF determinant to an excited Slater determinant or an entangled state involving two excited Slater determinants, as discussed above. Thus, unlike qEOM and QSE, where the excitation operators are measured together with the Hamiltonian, the excitation operators act directly on the HF state in q-sc-EOM. This provides a notable advantage that q-sc-EOM methods do not need the estimation of higher than 2-body RDMs. Such a framework also makes it easier to include higher excitations when required, such as triples, whose inclusion becomes important in higher-accuracy charged-state calculations.^89^ The calculation of elements of the **M** matrix can also be run in parallel on a quantum computer. The evaluation of the **M** matrix on a quantum computer requires the measurement of 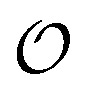 (*o*^4^*v*^4^) matrix elements, where *o* and *v* are the numbers of occupied and unoccupied orbitals, respectively. Despite this scaling, generally, the matrix **M** is very sparse. The number of elements in the **M** matrix can be drastically reduced using this sparsity through the use of spatial symmetry, spin symmetry, *etc.*, which will be a topic of future study. The major advantages afforded by the use of quantum computers come from the efficient evaluation of the q-sc-EOM matrix elements and from the accurate ground state wavefunction provided by the VQE-based algorithm.

## 3. Computational details

All the computations in this work utilize the STO-3G basis set. One- and two-electron integrals are calculated using the PySCF^90^ program with the HF reference state. The Jordan–Wigner mapping and the transformation of the second-quantized operators into the Pauli form are carried out using the OpenFermion^91^ software package. A classical noise-free simulator, where exact unitary operations are applied to the state vector representing the wavefunction, is used to assess the accuracy of the formalism developed in this work. The ground-state wavefunction is calculated using the fermionic ADAPT-VQE method using the generalized singles and doubles (GSD) operator pool.^11^ We use the gradient convergence criterion with a threshold of 10^−3^ for all the ground-state energy calculations. All the formalisms discussed in this work, namely q-sc-EOM, qEOM, and QSE, utilize the ground-state energy and wavefunction obtained from the ADAPT-VQE simulation. It should be noted that we have extended the qEOM formulation of ref. 75, originally developed for the calculation of EEs, to calculate IPs and EAs for our theoretical investigation. The EE results obtained using the qEOM approach in this work are verified against those of Qiskit's qEOM implementation.^92^ The code used for generating the data in this work can be found in ref. 93.

## 4. Results and discussion

We test the accuracy of the q-sc-EOM approach for three small molecules: H_2_, LiH, and H_2_O, and compare the results obtained with the exact FCI values. Computations are carried out for EEs, IPs, and EAs for these molecules. The total energies of the electronically excited, single electron-detached and single electron-attached states are computed by adding the EEs, IPs and EAs, calculated using q-sc-EOM, to the ground-state energy obtained using the ADAPT-VQE simulations. Since the ground-state energy computed using ADAPT-VQE is near-exact for the molecules considered in this study, the errors in the energies of the electronically excited states and the single electron attached/detached states, with respect to the FCI, are almost entirely due to the error in the post-VQE procedure. For LiH and H_2_O, we invoke the frozen-core approximation. Thus, the number of qubits required for the q-sc-EOM computation for H_2_, LiH, and H_2_O are 4, 10 and 12, respectively. We plot energy errors relative to the FCI values and use shading to indicate errors below 0.1 eV, as this value is generally the desired accuracy for these properties, so that they can be quantitatively compared with those in experiments. We also compare the performance of the q-sc-EOM formalism with that of QSE and qEOM in Subsection 4.2.

### 4.1 EE, IP, and EA calculations with q-sc-EOM

**Fig. 2:**
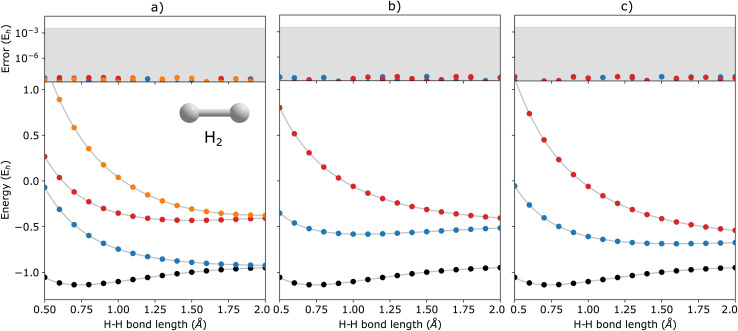
q-sc-EOM energies of (a) electronically excited, (b) single electron-detached, and (c) single electron-attached states along with the ground state (black circles) of the H_2_ molecule as a function of bond length. The gray lines correspond to the FCI results. The deviations from the FCI results are shown in the upper panel, where the shaded region indicates errors below 0.1 eV.

**Fig. 3:**
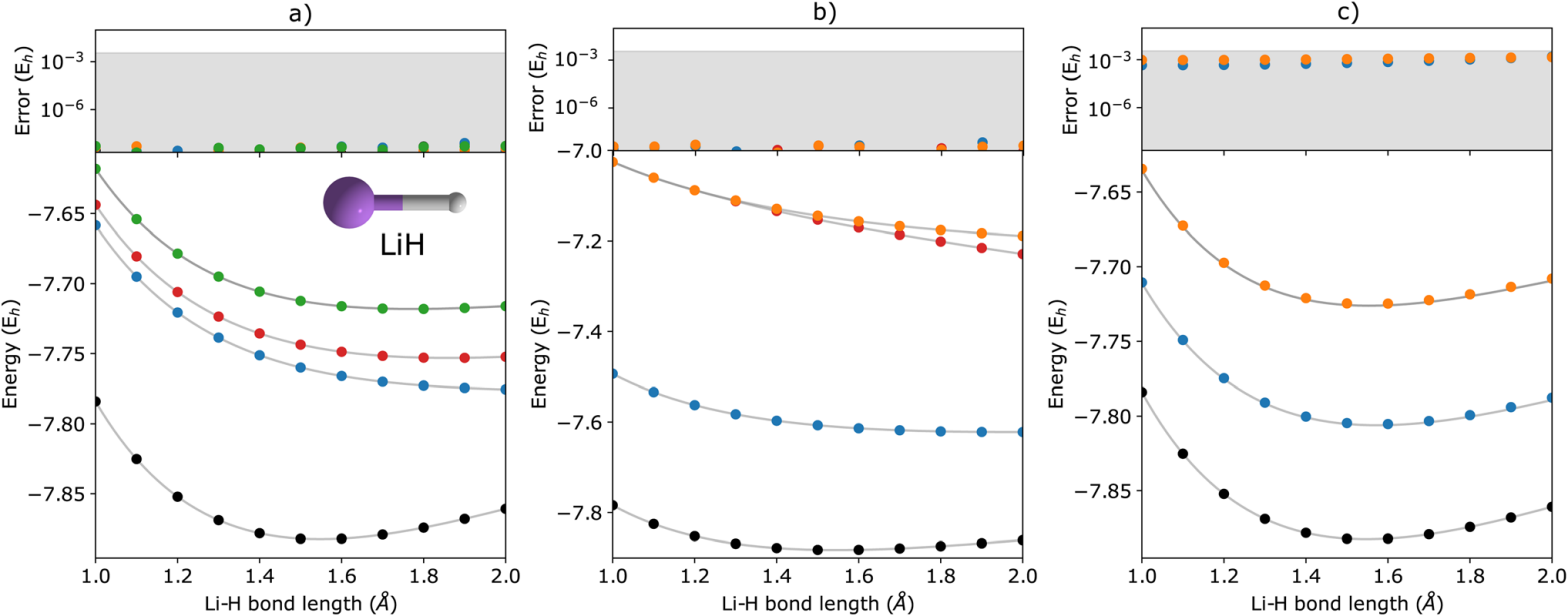
q-sc-EOM energies of the first few (a) electronically excited, (b) single electron-detached, and (c) single electron-attached states along with the ground state (black circles) of the LiH molecule as a function of bond length. The gray lines correspond to the FCI results. The deviations from the FCI results are shown in the upper panel, where the shaded region indicates errors below 0.1 eV.

**Fig. 4:**
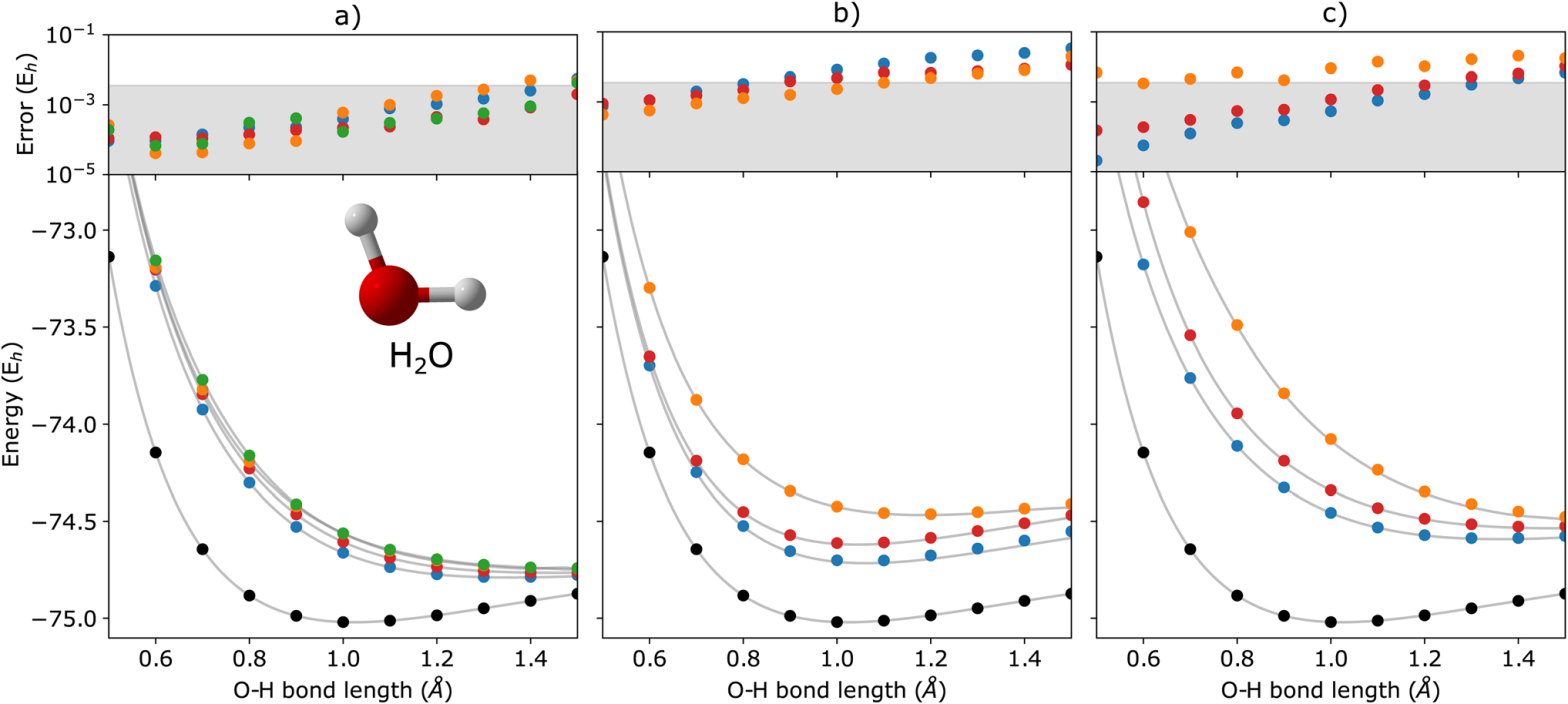
q-sc-EOM energies of the first few (a) electronically excited, (b) single electron-detached, and (c) single electron-attached states along with the ground state (black circles) of the H_2_O molecule as a function of the O–H bond lengths where both O–H bonds are stretched symmetrically. The gray lines correspond to the FCI results. The deviations from the FCI results are shown in the upper panel, where the shaded region indicates errors below 0.1 eV.

In Fig. 2, we show the energies of the ground state along with the first few electronically excited, single electron-detached and single electron-attached states of the H_2_ molecule calculated using q-sc-EOM, as a function of the inter-hydrogen distance. The corresponding FCI results are shown as gray lines. The errors in the energies with respect to the exact FCI values are shown in the subgraph on top of each panel. It can be seen that the errors in q-sc-EOM computed energies, or in other words, q-sc-EOM evaluated EEs, IPs, and EAs are essentially identical to those in FCI, with errors of less than 10^−8^ Hartree. This is because the q-sc-EOM formalism for the H_2_ molecule using the STO-3G basis set is exact, as the singles and doubles excitations used in eqn (19) span the full excitation space and the VAC is satisfied. Fig. 3 shows the energies of the ground state along with the first few electronically excited, single electron-detached and single electron-attached states for the LiH molecule as a function of the Li–H bond length in a similar layout as the previous figure. For both EEs and IPs, the q-sc-EOM method gives nearly exact results, and as expected, errors less than 10^−8^ Hartree were obtained with respect to the FCI values. However, we start to see the appearance of non-negligible errors in the EA results. This is because the computation of EAs for the LiH molecule with q-sc-EOM is no longer exact due to the addition of an electron. Thus, triple excitation operators should be added to the excitation manifold for an exact treatment for EAs. However, q-sc-EOM is still able to produce EAs within the desired accuracy, as seen from the shaded region in the error plot at the top of Fig. 3c. For the H_2_O molecule, we investigate the performance of q-sc-EOM as a function of the O–H bond length where both O–H bonds are stretched symmetrically. From Fig. 4, one can see that the errors in EEs are within the desired accuracy up to an O–H bond length of 1.4 Å. The errors build up as the two O–H bonds are broken further, due to the appearance of strong correlation effects in the wavefunction. Classical EOM-based methods, such as EOM-CCSD, show similar trends in errors as well. The errors in IPs and EAs are larger compared to those in EEs and are above our desirable error limit of 0.1 eV. This is well-known in classical quantum chemistry, where EOM-based methods require at least an approximate treatment of triple excitations in the EOM framework to reach an accuracy of 0.1 eV relative to FCI values for IPs and EAs.^89,94,95^ For higher accuracy in charged excitations, carefully selected triple excitations can be added to the excitation manifold in q-sc-EOM. This will be a subject of future study.

### 4.2 Comparison with QSE and qEOM

In this subsection, we discuss the connections between the q-sc-EOM, QSE, and qEOM formalisms and compare them in the contexts of (a) their accuracy in computing energy differences and (b) quantum resource requirements and sensitivity to noise. The EOM formulation in eqn (15) with excitation operators taken from the excitation manifold represented as {*Ĝ*^†^_*I*_}∪{*Ĝ*_*I*_} (not the self-consistent excitation manifold) may lead to a violation of the VAC. There are two methods discussed in the quantum chemistry literature to impose the VAC in the EOM formalism: the projected operator approach (see ref. 79) and the self-consistent operator formalism (see ref. 78). If we use projected operators based on ref. 79, we arrive at a QSE-type formalism (as performed in ref. 96) to calculate excited-state properties. The q-sc-EOM formalism developed in this work is based on the use of self-consistent excitation operators to impose the VAC. It should be noted that this concept has been utilized in the development of different excited-state methods in classical quantum chemistry.^85,97–99^

#### 4.2.1 Accuracy of energy differences

**Fig. 5:**
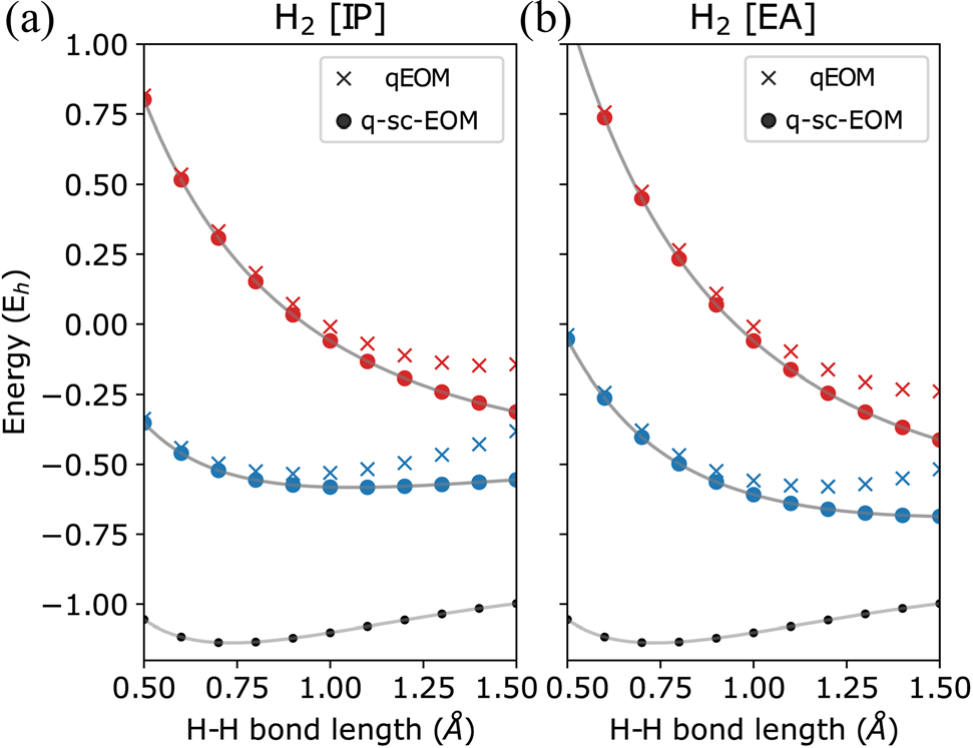
Energies of (a) single electron-detached and (b) single electron-attached states along with the ground state (black circles) of the H_2_ molecule plotted as a function of the H–H bond length using the STO-3G basis. The FCI results are plotted in gray.

**Fig. 6:**
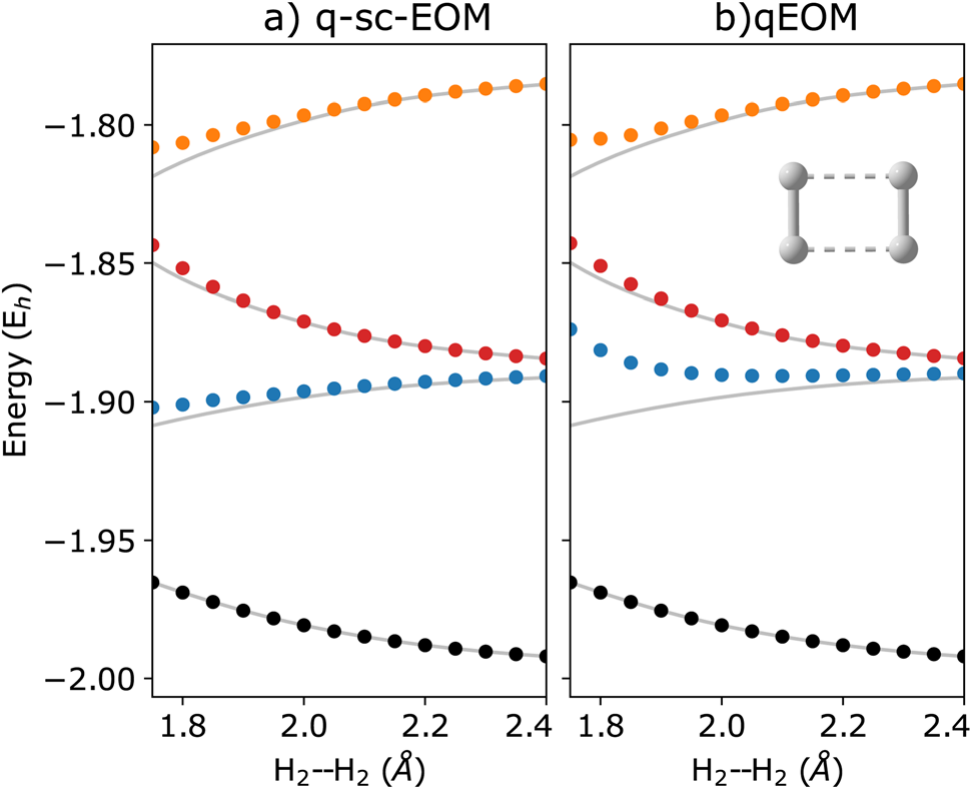
Energies of the three lowest excited states along with the ground state (black circles) computed using (a) q-sc-EOM and (b) qEOM formalisms for the dissociation of a rectangular geometry of H_4_ into H_2_⋯H_2_ as a function of the H_2_⋯H_2_ separation distance. Both of the H_2_ molecules have a bond length of 1.5 Å. The FCI results are plotted in gray.

The qEOM method, which uses the EOM formulation in eqn (16) with the conventional excitation manifold, {*Ĝ*^†^_*I*_}∪{*Ĝ*_*I*_} (details can be found in ref. 75), may lead to large errors in calculated IPs and EAs due to the violation of the VAC. This can be seen through the qEOM-evaluated IPs and EAs added to the almost exact ADAPT-VQE ground state of the H_2_ molecule in the STO-3G basis as shown in Fig. 5. Large deviation from the FCI results can be observed in this image. It should be noted that although single and double excitations span all the possible excitations in the case of the H_2_ molecule in the STO-3G basis, the VAC is still not satisfied in qEOM. This is because the excitation manifold is not complete with respect to the exact ground-state when we use the {*Ĝ*^†^_*I*_}∪{*Ĝ*_*I*_} operator manifold corresponding to electron detached/atached states. One way to solve this issue is by increasing the size of the operator manifold. However, this would significantly increase the computational cost. Fig. 6 shows the energies of the three lowest excited states evaluated using qEOM and q-sc-EOM methods for a rectangle geometry H_4_ molecular system as a function of H_2_⋯H_2_ separation distance. The H–H bond distance is fixed at 1.5 Å in the two H_2_ molecules. We observe from Fig. 6 that q-sc-EOM is more robust in strongly correlated situations, compared with the qEOM method. The first and third excited states of H_4_ computed using the qEOM method show qualitatively wrong behaviour in the region shown in the figure.

**Fig. 7:**
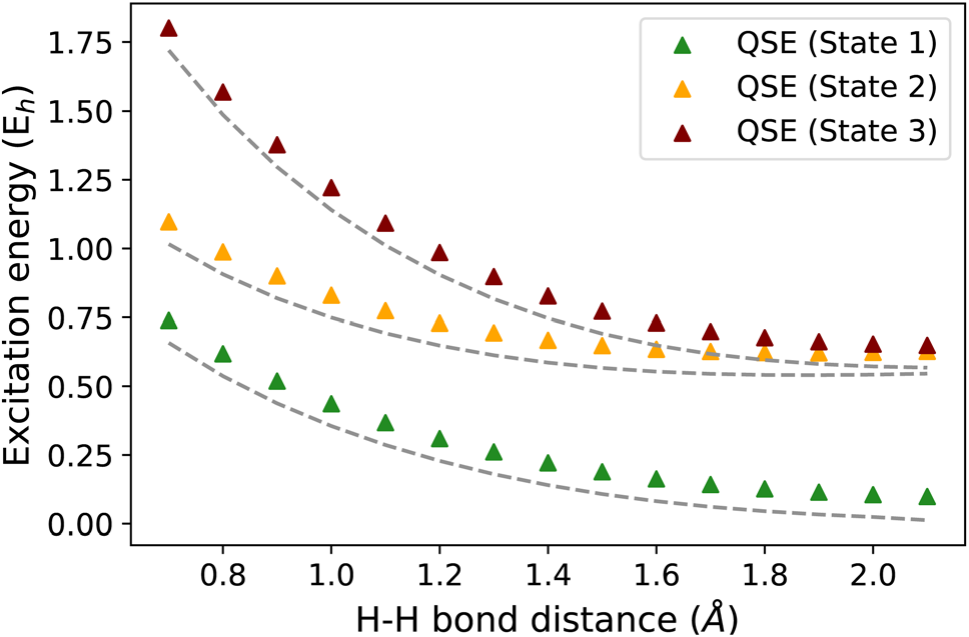
Excitation energies of the H_2_ molecule in isolated H_2_ (grey lines) and an H_2_–H_4_ system with H_2_ not interacting with H_4_ (H_2_ and H_4_ separation taken as 100 Å) (triangles) using the STO-3G basis set. An inexact ground state is used in the plot computed using ADAPT-VQE stopped after adding 3 operators. The difference in the dashed line with triangles represents the size-intensivity error for each excitation energy using QSE.

The EEs obtained using QSE are not necessarily size-intensive for an inexact ground state. This is due to the inclusion of an identity operator in the operator manifold used in QSE. We illustrate this point using an H_2_⋯H_4_ molecular system as an example. Fig. 7 shows the difference in EEs computed for an isolated H_2_ molecule and an H_2_⋯H_4_ molecular supersystem with no interaction between the H_2_ and H_4_ subsystems (the distance between H_2_ and H_4_ is taken as 100 Å). An inexact ground state is taken using an ADAPT-VQE simulation that is stopped after adding 3 operators. An identity operator is added to the operator manifold of QSE which uses the operator manifold represented by {*Ĝ*_*I*_} in Section 2.1. QSE computations give an error of ∼81 mH in this test, while the q-sc-EOM method shows the correct behavior. In this scenario, the two EEs should be identical for a method that provides size-intensive EEs. The magnitude of this size-intensivity error in QSE will depend on the accuracy of the ground state. Since it is expected that near-term quantum computers may not provide exact ground-state energies for all molecular cases, these size-intensivity errors may cause problems. We note here that, just like q-sc-EOM, the qEOM method provides size-intensive EEs, IPs and EAs as well.

#### 4.2.2 Noise-sensitivity

Along with the above mentioned theoretical benefits, q-sc-EOM also has important computational advantages. q-sc-EOM is expected to be more noise-resilient compared with qEOM and QSE. A prime reason for this is because q-sc-EOM requires up to 2-RDM type measurements while QSE and qEOM require measurement of higher-body RDMs. Estimation of higher-body RDMs can significantly increase the noise in measured matrix elements. This has been noted in ref. 100 and shown using simple noise models that the error in the expectation value will scale by a factor exponential in the number of qubits measured together. q-sc-EOM strictly requires measurement of up to 2-RDMs or 4 qubit measurements, while QSE and qEOM require measurements of up to 12 qubits at a time.

Additionally, we have also carried out a theoretical study based on matrix perturbation theory to compare the noise-resilience of QSE with q-sc-EOM on a noisy quantum computer. The analysis is carried out for an interacting pair of H_2_ molecules (H_4_ structure with a rectangular geometry with 1.5 Å and 2.0 Å bond distances). We model the effect of noise by adding random errors directly to each matrix element utilized by the two methods. It should be noted that in real implementations where these matrix elements are measured on quantum computers, there will be multiple sources of errors (gate errors, measurement errors, *etc.*), which will finally create a net error in each matrix element. In our noise study, it is reasonable to add errors directly as we assume that all matrix elements, both in QSE and q-sc-EOM, will have errors of the same magnitude when the same quantum resources are used. This is an appropriate (and rather conservative) assumption for two reasons: first, all measurements in both q-sc-EOM and QSE utilize the same ground-state quantum circuit (with the difference being that in q-sc-EOM the circuit is applied on different reference states). Second, q-sc-EOM makes use of at most 2-body RDMs, while QSE requires the estimation of higher-body RDMs as well, which is expected to generate errors of the same or higher magnitude in the case of QSE. A more detailed noise analysis performed on an actual quantum device will be the subject of future studies.

**Fig. 8:**
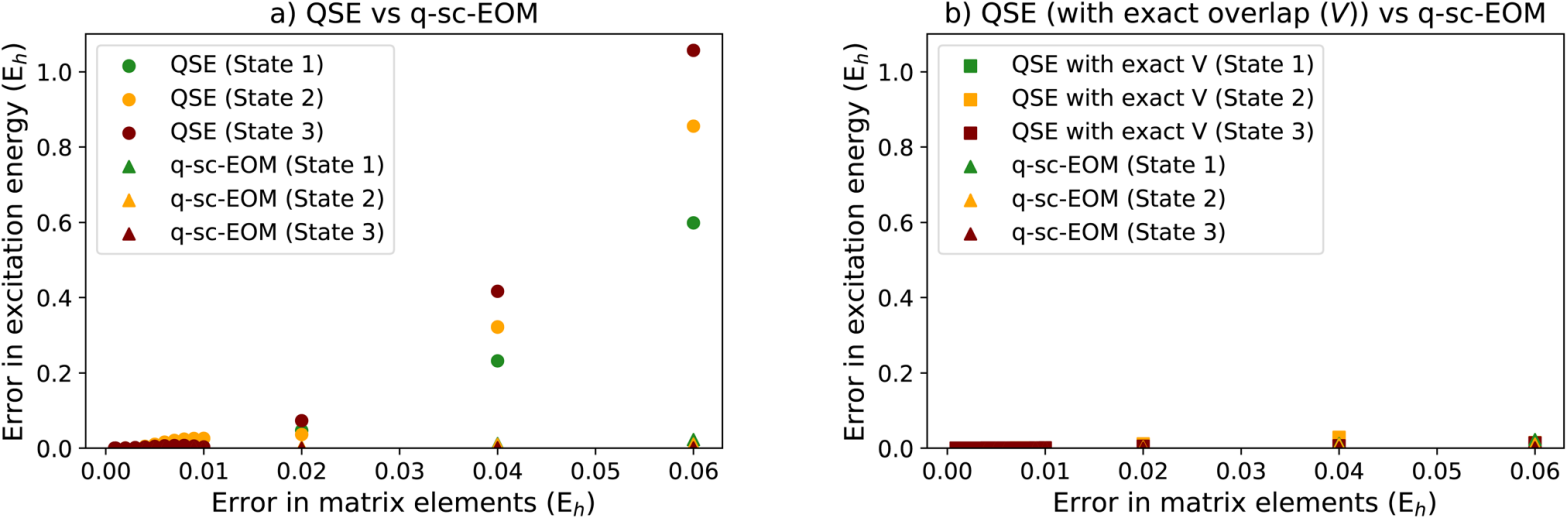
(a) Error in the first few excitation energies computed for H_4_ using the QSE *versus* q-sc-EOM frameworks plotted against the maximum magnitude of errors in the matrix elements before diagonalization. (b) Error in the first few excitation energies computed for H_4_ using the QSE (with no error in the overlap matrix (*V*) *versus* q-sc-EOM frameworks plotted against the maximum magnitude of errors in the matrix elements before diagonalization. A rectangle geometry H_4_ molecular system is used for the calculations with 1.5 Å and 2.0 Å as H–H distances. This shows that most of the noise sensitivity in QSE arises from noise in the computed overlap matrix, while the overlap matrix in q-sc-EOM is exactly the identity matrix and thus noise-free.

In Fig. 8a, we show the performance of QSE compared with that of q-sc-EOM, where to each matrix element we add random offsets sampled from a uniform distribution in line with matrix perturbation theory. The horizontal axes in the figure correspond to the upper bound of this distribution (*i.e.*, the maximum allowed error). The EEs are calculated after solving the eigenvalue equation of q-sc-EOM and the generalized eigenvalue equation of QSE (the latter resembles eqn (18)). Errors are defined with respect to the EE values obtained from the associated method in the absence of noise. Fig. 8a shows the error averaged over 100 000 calculations of the EEs with different random offsets in each case. We can observe in the figure that QSE produces much larger errors at the same level of noise compared to q-sc-EOM, thus showing that we can expect q-sc-EOM to be more resilient to noise than QSE.

A key difference to consider between QSE and q-sc-EOM is that the overlap matrix **V** in q-sc-EOM is exactly known to be the identity matrix and thus does not need to be measured, while in the case of QSE, the overlap matrix must be measured on a quantum computer. Note that the latter is also true for qEOM. Fig. 8b shows that if the overlap matrix is exactly known in QSE (*i.e.*, we compute it exactly without noise), the noise-resilience of QSE is similar to that of q-sc-EOM. This suggests that the knowledge of the exact overlap matrix in q-sc-EOM is critical in providing noise-resilience, whereas methods that measure the overlap matrix and thus solve the generalized eigenvalue problem (such as QSE and qEOM) are expected to be more sensitive to noise. This noise-sensitivity, we believe, is a direct result of a noise-sensitive matrix inversion step in solving a generalized eigenvalue problem. A detailed analysis of this problem for general quantum algorithms for ground and excited state estimation will be presented in a future work.

## 5. Conclusion

In this work, we propose a new method, named q-sc-EOM, for calculating molecular excitation energies using a quantum computer. The method can be implemented on top of any quantum variational algorithm used to obtain the ground state of the target molecule. Our approach is inspired by excited-state methods developed in quantum chemistry, specifically the ones based on the UCC theory. q-sc-EOM has several important benefits compared to current state-of-the-art excited-state methods for the NISQ era, with theoretical benefits including: (a) q-sc-EOM uses self-consistent operators that satisfy the vacuum annihilation condition, and thus it can be generalized to evaluate accurate vertical excitation energies, ionization potentials, and electron affinities; (b) energy differences obtained using q-sc-EOM are strictly size-intensive, an important property that ensures their correct scaling with the size of the molecular system; (c) q-sc-EOM is a Hermitian theory, providing guaranteed real energy differences. The major computational benefit of q-sc-EOM is that it can be expected to be more resilient to noise because (a) q-sc-EOM does not require higher than 2-body RDMs; and (b) it requires a classical solution of the eigenvalue equation, bypassing the noise-sensitive step associated inversion of the overlap matrix in solving the generalized eigenvalue equation in QSE and qEOM. These benefits provide important theoretical and practical advantages for the computation of excitation energies on near-term quantum devices.

NISQ era devices are expected to be noisy with limited resources. Thus, to achieve an advantage through these devices in quantum chemistry problems over classical computation, one should use methods that are meaningfully accurate, while at the same time being resistant to errors and resource-efficient. The q-sc-EOM method proposed in this work is promising in this regard because it exhibits many of the crucial properties of highly successful EOM-based quantum chemistry methods. At the same time, it remains resource-efficient and is expected to be resilient to noise compared to the currently available diagonalization-based methods. Our future studies will combine q-sc-EOM with the recently developed transcorrelated Hamiltonian formalism^101,102^ to obtain quantitatively accurate excited-state properties with minimal utilization of quantum resources, which otherwise generally requires the use of large basis sets.

## Author contributions

A. A. and A. K.: conceptualization, writing – original draft; N. J. M., A. A., A. K., and V. A.: software, formal analysis, V. A.: validation; H. G.: software, methodology; N. J. M., S. E. E., E. B., Y. Z., L. C., S. T., and P. A. D.: supervision, project administration, funding acquisition; all authors: writing – review & editing.

## Conflicts of interest

There are no conflicts to declare.

## Supplementary Material
